# ﻿Three new species of *Clavariadelphus* (Clavariadelphaceae, Gomphales) from the Hengduan Mountains region, China

**DOI:** 10.3897/mycokeys.121.158142

**Published:** 2025-09-02

**Authors:** Mei-jia Li, Peng-tao Deng, Zuo-hong Chen, Ping Zhang

**Affiliations:** 1 College of Life Science, Hunan Normal University, Changsha 410081, China Hunan Normal University Changsha China

**Keywords:** Club-shaped fungi, morphological characteristics, new taxa, phylogenetic analysis, taxonomy

## Abstract

Specimens of three unnamed club-shaped fungi, collected from the Hengduan Mountains region in China, are formally described as *Clavariadelphus acuminatus*, *C. miniatus*, and *C. pseudoelongatus*. Based on ITS, nrLSU, ATP6, and TEF1 sequence data, a multigene phylogenetic analysis indicated that the three new species each formed a distinct lineage within the *Clavariadelphus* clade with strong support. Descriptions, illustrations, and the results of molecular phylogenetic analyses for the three newly discovered species are presented. A taxonomic key for the known *Clavariadelphus* species in China is provided.

## ﻿Introduction

*Clavariadelphus* Donk (Clavariadelphaceae, Gomphales, Basidiomycota) is a genus of symbiotic or saprophytic clavarioid fungi (Deng et al. 2020). Some members of the genus are reported to be mycorrhizal with diverse hosts, such as oak, pine, and spruce species (Hanif et al. 2022). The genus was initially proposed in 1933 to accommodate three species, comprising *C. pistillaris* (L.) Donk, *C. ligula* (Schaeff.) Donk, and *C. truncatus* (Quél.) Donk, with *C. pistillaris* designated as the type species (Corner 1950). The genus includes unbranched clavarioid fungi with simple, erect, club-shaped basidiomata; a thick hymenium composed of (2–)4-spored basidia and clavate leptocystidia; ellipsoid to amygdaliform, smooth, thin-walled, inamyloid basidiospores; and clamped tramal hyphae (Corner 1950, 1970; Methven 1990). The spore length-to-width ratio is an important distinguishing character for *Clavariadelphus*. The genus was divided into sect. Clavariadelphus and sect. Ligula based on the spore length-to-width ratio (Wells and Kempton 1968). The spores of some *Clavariadelphus* species are ellipsoid to broadly ellipsoid (*Q*_m_ < 2.5), as in sect. Clavariadelphus, whereas in other species the spores are narrowly ellipsoid (*Q*_m_ ≥ 3), as in sect. Ligula (Wells and Kempton 1968).

*Clavariadelphus* has a widespread distribution in temperate forests of the Northern Hemisphere. The majority of early-described *Clavariadelphus* species were reported from Europe and North America. In *Index Fungorum* (Partnership 2010), 47 fungal records have been assigned to *Clavariadelphus*, of which 33 species are formally accepted (Deng et al. 2020; Huang et al. 2020; Xia and Fan 2020), and the remaining records are classified in other fungal groups. In 2020, seven *Clavariadelphus* species had been recorded exclusively from China, namely, *C. alpinus* J. Zhao & L.P. Tang, *C. amplus* J. Zhao, L.P. Tang & Z.W. Ge, *C. aurantiacus* P. Zhang, *C. gansuensis* J. Zhao & L.P. Tang, *C. griseoclavus* L. Fan & L. Xia, *C. khinganensis* J. Zhao, L.P. Tang & P. Zhang, and *C. tenuis* P. Zhang.

The Hengduan Mountains region in China is one of 34 global biodiversity hotspots (Myers et al. 2000; Mittermeier et al. 2005). Previously, the species diversity of macrofungi from the Hengduan Mountains region has been investigated, and many new species have been discovered from the region (Cui et al. 2018; Dai et al. 2021; Ji et al. 2022; Liu et al. 2023; Song et al. 2025). In the present study, using our collections of *Clavariadelphus* specimens, three new *Clavariadelphus* species from the Hengduan Mountains region are identified, which are described herein as *C. acuminatus*, *C. miniatus*, and *C. pseudoelongatus*.

## ﻿Materials and methods

### ﻿Fungal material

Three new species of *Clavariadelphus* were collected from the Hengduan Mountains region during 2018–2024. The fresh basidiomata were photographed and recorded relevant characteristics in the field. All collected specimens were dried at 50 °C–60 °C in a drying oven or desiccated in silica gel. The dried specimens are housed in the Mycological Herbarium of Hunan Normal University (MHHNU), Changsha, China.

### ﻿Morphological descriptions

Macromorphological characters were described from detailed field notes and habitat photographs. A section of dried hymenial tissue was taken from the materials and placed in 5% KOH solution containing 1% Congo Red or Melzer’s reagent. The microscopic structure of each species was measured as at least 25 sets of parallel data, including the basidia, basidiospores and hyphae. Color descriptions and codes follow Kornerup and Wanscher (1978) and Ridgway (1912). In the basidiospore description, the size of basidiospores is expressed in the form (a–) b–c (–d), where the range b–c is the majority of measured values, and *a* and *d* are extreme values of the spore dimension. The notation [n/m/p] refers to the number of measurements recorded for n basidiospores from m basidiomata of p specimens. The *Q* value represents the length-to-width ratio of basidiospores, and the *Q_m_* value is the average *Q* ± standard deviation.

### ﻿Scanning electron microscopy

A small portion of tissue containing the hymenium was sampled from the dried specimens, mounted onto aluminum stubs, and coated with gold palladium. Scanning electron microscopy was conducted with a TESCAN CLARA Xplore 30 operating at 20 keV. The scanning electron micrographs of basidiospores of three new *Clavariadelphus* species were shown in Fig. 2.

### ﻿DNA extraction, PCR amplification, and sequencing

Total genomic DNA was extracted from the collected specimens using the Ezup Column Fungi Genomic DNA Purification Kit (Sangon Biotech, Shanghai, China). The universal primers ITS4/ITS5 and LR5/LR0R were used to amplify the internal transcribed spacer (ITS) region of ribosomal DNA and the nuclear large subunit (nrLSU) region in the 28S ribosomal RNA gene (Vilgalys and Hester 1990; White et al. 1990; Gardes and Bruns 1993). The *ATP6* and *TEF1* genes were respectively amplified using the primer pairs ATP6-1R/ATP6-1F and EF-CR/EF-CF or EF-d2/EF-d1 (Deng 2021). Each PCR amplification reaction was performed on an Eppendorf Mastercycler thermal cycler (Eppendorf, Inc., Hamburg, Germany). The 25 µL reaction mixture contained 1 × PCR buffer, 1.5 mM MgCl_2_, 0.2 mM dNTP_S_, 0.4 μM of each primer, 1.25 U Taq polymerase (Sangon Biotech), and 1 μL DNA template. The PCR protocol for each amplification was as follows: pre-denaturation at 94 °C for 5 min, then 34 cycles of denaturation at 94 °C for 30 s, annealing at an appropriate temperature (55 °C for 30 s for ITS and nrLSU, 52–54 °C for 40 s for *TEF1* and *ATP6*; He et al. 2023), and extension at 72 °C for 1 min, followed by a final extension at 72 °C for 7 min, and then held at 8 °C. The amplified PCR products were separated by 1% agarose gel electrophoresis and the same primers were used for sequencing by Sangon Biotech. All sequences newly generated in this study were deposited in GenBank (accession numbers are listed in Table 1).

**Table 1.** Details of sequences used and produced in phylogenetic analysis. The sequences newly generated in this study are highlighted in bold. The type specimens and type species in *Clavariadelphus* are indicated with asterisks (*); – represents data unavailability.

Taxon	Voucher	GenBank No. ITS	GenBank No. nrLSU	GenBank No. TEF1	GenBank No. ATP6	Location	References
Clavariadelphus acuminatus	MHHNU 12163*	PV463549	PV490621	–	PV523761	China	This study
C. acuminatus	MHHNU 12164	PV463550	PV490622	–	PV523762	China	This study
*C. americanus*	MycoMap # 1288	MK575228	–	–	–	USA	unpublished
*C. amplus*	HKAS 76577	MK705851	MK704443	MK736675	–	China	Huang et al. (2020)
*C. amplus*	HKAS 54876*	MK705857	MK704444	MK736676	–	China	Huang et al. (2020)
*C. amplus*	HKAS 49229	MK705854	MK704447	MK736677	–	China	Huang et al. (2020)
*C. amplus*	HMAS 250466	MK705858	MK704448	–	–	China	Huang et al. (2020)
*C. aurantiacus*	MHHNU 9256*	MT580787	PV490611	PV523772	PV523753	China	Deng et al. (2020), this study
*C. aurantiacus*	MHHNU 10085	MT580792	PV490612	PV523773	PV523754	China	Deng et al. (2020), this study
*C. aurantiacus*	HKAS 53889	MT580788	–	–	–	China	Deng et al. (2020)
*C. elongates*	SWAT000559	MG768848	–	–	–	Pakistan	Sher et al. (2018)
*C. elongates*	LAH31397	MG768847	–	–	–	Pakistan	Sher et al. (2018)
*C. elongates*	HMAS 260746	MK705845	MK704441	MK736672	–	China	Huang et al. (2020)
*C. elongates*	HKAS 50742	MK705843	MK704440	–	–	China	Huang et al. (2020)
*C. elongates*	MHHNU 9250	PV463551	PV490604	PV523767	PV523746	China	This study
*C. elongates*	MHHNU 9933	PV463552	PV490605	PV523768	PV523747	China	This study
*C. griseoclavus*	BJTC FM964	MT302370	–	–	–	China	Xia and Fan (2020)
*C. griseoclavus*	BJTC FM965	MT302371	–	–	–	China	Xia and Fan (2020)
*C. gansuensis*	HKAS 76487	MK705847	MK704442	MK736673	–	China	Huang et al. (2020)
*C. himalayensis*	HKAS 50684	MK705863	–	–	–	China	Huang et al. (2020)
*C. himalayensis*	HKAS 58811	MK705864	–	–	–	China	Huang et al. (2020)
*C. himalayensis*	MHHNU 9167	PV463553	PV490610	PV523771	PV523750	China	This study
*C. khinganensis*	MHHNU 7789*	MK705865	MK704451	MK736680	PV523758	China	Huang et al. (2020), this study
*C. khinganensis*	MHKMU H.Y. Huang 368	MT447468	–	–	–	China	Huang et al. (2020)
*C. khinganensis*	MHHNU 7822	PV463554	PV490616	PV523776	PV523759	China	This study
*C. ligula*	OMDL iNat # 180557549	PP959633	–	–	–	USA	Unpublished
*C. ligula*	NAMPA2215-22	OP225575	–	–	–	USA	Unpublished
*C. ligula*	AMB 18570	MT055950	–	–	–	Italy	Unpublished
*C. ligula*	3833	KM248918	–	–	–	Canada	Unpublished
*C. ligula*	MHHNU 10452	PV463555	PV490608	–	PV523751	China	This study
*C. ligula*	MHHNU 10453	PV463556	PV490609	–	PV523752	China	This study
*C. mucronatus*	OSC 1064138	EU526000	–	–	–	USA	Smith et al. (2002)
C. miniatus	MHHNU 9952*	PV463557	PV490619	PV523777	PV523760	China	This study
C. miniatus	MHHNU 12089	PV463558	PV490620	–	–	China	This study
*C. occidentalis*	OSC 104664	EU669308	–	–	–	USA	Unpublished
*C. occidentalis*	OSC 114281	EU846242	–	–	–	USA	Unpublished
*C. pakistanicus*	mh129901*	HQ379937	–	–	–	Pakistan	Hanif et al. (2014)
*C. pakistanicus*	MHHNU 9282	MT580789	PV490603	PV523766	PV523745	China	Deng et al. (2020), this study
C. pistillaris*	NAMA 2017-123	MH979250	–	–	–	USA	Unpublished
C. pistillaris*	FLAS-F-60521	MH281842	–	–	–	USA	Unpublished
C. pseudoelongatus	MHHNU 32323*	PV463559	PV490617	–	–	China	This study
C. pseudoelongatus	MHHNU 12123	PV463560	PV490618	–	–	China	This study
*C. sachalinensis*	OSC 96213	EU834196	–	–	–	USA	Unpublished
*C. sachalinensis*	p059i	EU624410	–	–	–	USA	Unpublished
*C. sachalinensis*	MHHNU 7816	MT580791	MK704452	–	PV523744	China	Huang et al. (2020), this study
*C. sachalinensis*	p058i	EU624411	–	–	–	USA	Unpublished
*C. subfastigiatus*	OSC 119587	EU669207	EU669259	–	–	USA	Unpublished
*C. subfastigiatus*	MICH 73554	JX275756	–	–	–	USA	Unpublished
*C. truncates*	SIM278	HQ650728	–	–	–	Canada	Kranabetter et al. (2009)
*C. truncates*	QHU20413	OM974113	OM942741	–	–	China	Unpublished
*C. truncates*	UBC: F30993	MZ836052	–	–	–	Canada	Unpublished
*C. tenuis*	MHHNU 9897	MT580786	PV490613	PV523774	PV523755	China	Deng et al. (2020), this study
*C. tenuis*	MHHNU 9900	MT580793	PV490614	–	PV523756	China	Deng et al. (2020), this study
*C. tenuis*	MHHNU 9934*	MT580790	PV490615	PV523775	PV523757	China	Deng et al. (2020), this study
*C. unicolor*	Mushroom Observer # 112193	MN906166	–	–	–	USA	Unpublished
*C. yunnanensis*	HMAS 250510	MK705874	MK704458	MK736685	–	China	Huang et al. (2020)
*C. yunnanensis*	HKAS 54849	MK705869	MK704453	MK736681	–	China	Huang et al. (2020)
*C. yunnanensis*	HKAS 63558	MK705870	MK704454	MK736682	–	China	Huang et al. (2020)
*C. yunnanensis*	MHHNU 9244	PV463561	PV490606	PV523769	PV523748	China	This study
*C. yunnanensis*	MHHNU 9977	PV463562	PV490607	PV523770	PV523749	China	This study
*L. byssiseda*	MHHNU 10765	PV463563	PV490623	–	PV523763	China	This study
*L. byssiseda*	MHHNU 9087	PQ248145	PQ242649	–	PV523764	China	Li et al. (2025), this study
*L. patouillardii*	MHHNU 10443	PQ248147	PQ242651	–	PV523765	China	Li et al. (2025), this study
*L. patouillardii*	HMJAU 26892	KU870449	–	–	–	China	Liu et al. (2017)
Gomphus clavatus*	EL 64/03	EU118628	–	–	–	Sweden	Larsson (2007)
*G. ludovicianus*	TFB14476 clone c8*	KJ655570	–	–	–	USA	Petersen et al. (2014)

### ﻿Alignment and phylogenetic analysis

In addition to the newly generated sequences from 25 specimens, the remainder of the sequences used in this study were publicly available from GenBank. A multiple sequence alignment for each of the four gene fragments, namely, ITS, nrLSU, *TEF1*, and *ATP6*, was constructed with MAFFT v7.149 (Katoh and Standley 2016). The aligned sequences were manually adjusted and trimmed as necessary with BioEdit v7.2.5 (Hall 1999). A combined matrix, comprising 146 sequences, was generated with Sequence Matrix v7.2.5 (Hall 1999). Two species of *Gomphus* (Pers.) Gray, *G. clavatus* (Pers.) Gray and *G. ludovicianus* R.H. Petersen, Justice *&* D.P. Lewis, were selected as the outgroup. Maximum likelihood analysis was conducted with RAxML v7.2.6 (Stamatakis et al. 2005; Stamatakis 2006), using a GTR+Gamma evolutionary model (Stamatakis et al. 2008) with 1000 bootstrap replicates. Bayesian inference (BI) was performed using MrBayes v3.2.7 (Ronquist et al. 2012) with the GTR+I+G optimal evolutionary model, and analyses were run for 2,000,000 generations using four Metropolis-coupled Monte Carlo Markov chains to calculate posterior probabilities (PP). The tree files were visualized with FigTree v1.4.2 (Rambaut 2012) and slightly adjusted for presentation purposes with Photoshop CS6 and Illustrator CS6 (Adobe Systems, Inc., San Jose, CA, USA).

## ﻿Results

### ﻿Phylogenetic analyses

**Figure 1.**
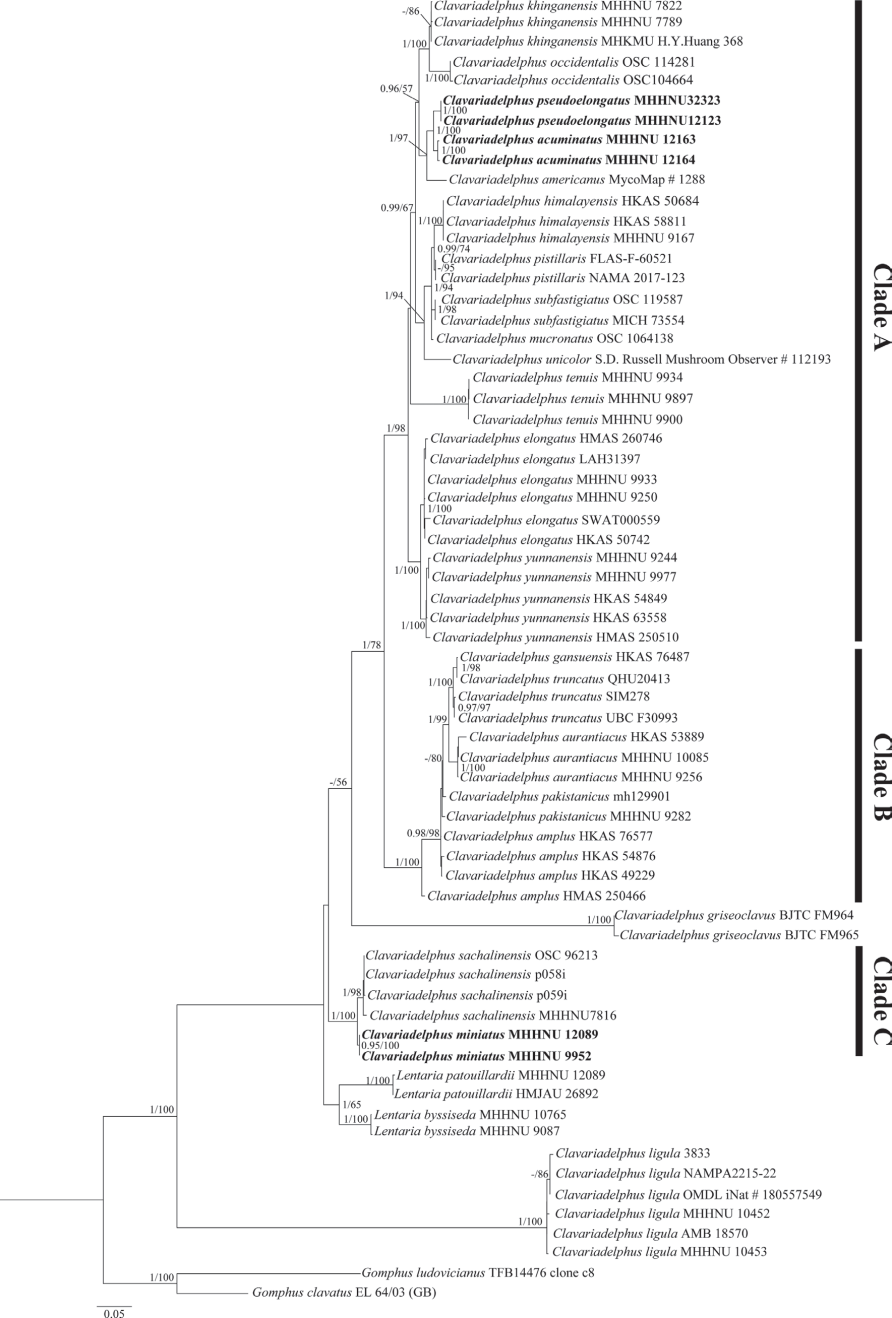
Maximum likelihood (ML) phylogenetic tree of *Clavariadelphus* species and *Lentaria* species inferred from the concatenated multigene sequence dataset (ITS, nrLSU, *ATP6*, and *TEF1*). The Bayesian inference posterior probabilities ≥ 0.95 and ML bootstrap values ≥ 50% are shown at the nodes; “–” indicates that the values were less than these thresholds. Accessions of the three new *Clavariadelphus* species are in bold.

The final multigene dataset, which constituted 146 sequences (66 ITS, 22 *ATP6*, 21 *TEF1*, and 37 nrLSU), was used for the ML and BI analyses. The matrix of concatenated sequences comprised 2776 bp. The phylogenetic tree included 22 *Clavariadelphus* taxa, 2 *Lentaria* Corner taxa, and two *Gomphus* species (*G. clavatus* and *G. ludovicianus*) as the outgroup. The ML tree (Fig. 1) was highly similar in topology to the BI tree (not shown). The bootstrap support (BS) values and posterior probabilities (PP) of relevant nodes, which were greater than or equal to 50% and 0.95, respectively, are shown in Fig. 1. In this study, six samples of three newly gathered *Clavariadelphus* species were placed in different *Clavariadelphus* subclades with strong support (all PP 1, BS 100%) and each formed a distinct lineage. *Clavariadelphus acuminatus* and *C. pseudoelongatus* were sister taxa within a well-supported subclade (PP 1, BS 100%). The novel saprophytic species *C. miniatus* formed a distinct lineage sister to another saprophytic species, *C. sachlinensis* (S. Imai) Corner, with strong statistical support (PP 1, BS 100%). The results of the molecular phylogenetic analyses supported the distinctness of the three putative new *Clavariadelphus* species, which are formally described herein.

**Figure 2.**
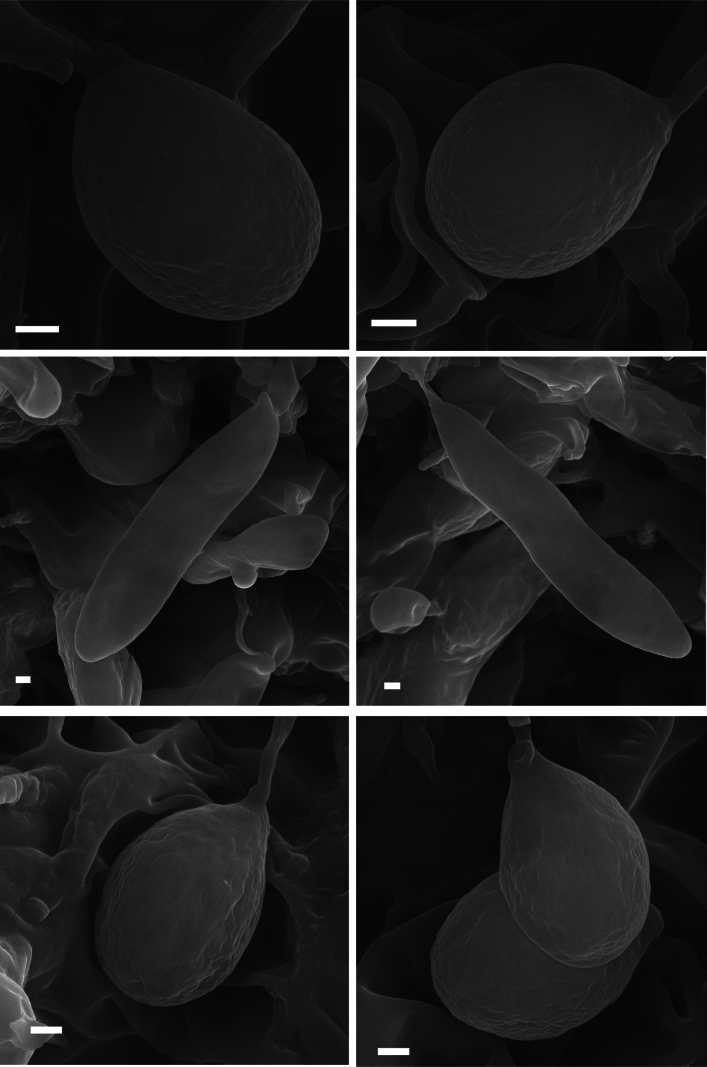
Scanning electron micrographs of basidiospores of the three new *Clavariadelphus* species. A, B. *C. acuminatus* (MHHNU 12163, holotype); C, D. *C. miniatus* (MHHNU 9952, holotype); E, F. *C. pseudoelongatus* (MHHNU 32323, holotype). Scale bars: 1 µm.

### ﻿Taxonomy

#### ﻿ Clavariadelphus acuminatus

Taxon classificationFungiGomphalesClavariadelphaceae

X.L. Gao & P. Zhang sp. nov.

12918818-73EB-5076-A1A7-92B3A1F77603

858803

##### Diagnosis.

Differs from other *Clavariadelphus* species in that the apex of the basidiomata is acuminate and pale to brownish yellow or golden-brown in color.

##### Type.

China • Yunnan Province, Lufeng County, 25°24'02"N, 101°76'10"E, 2300 m asl., 4 September 2023, leg. X.L. Gao (holotype MHHNU 12163).

##### Etymology.

*Acuminatus* (Latin) refers to the acuminate apex of the basidiomata.

##### Description.

***Basidiomata*** up to 15 cm high, 0.4–0.8 cm in diameter at the base, 0.8–1.2 cm in diameter in the middle, slightly tapering at both ends, simple, initially narrowly cylindrical, narrowly fusiform after maturity, mostly flexuous; ***hymenium*** initially smooth, then longitudinally rugose with age, pale (2A2) to pale orange (5A3) to apricot (5B6); ***apex*** fertile, obtuse to acute, smooth to rugose, darker than the hymenium with age, pale to brownish yellow (5C8) or golden brown (5D7); ***base*** terete, almost smooth, white to cream (4A3); ***context*** solid when young, gradually becoming soft and spongy with age. Odor and taste not recorded.

***Hymenium*** extending over the apex of the basidioma, composed of basidia and leptocystidia. ***Basidia*** 75–99 × 8.5–13 µm, narrowly clavate, pale yellow in KOH, smooth, thin-walled, clamped, 4 sterigmata (rarely 2), 6–12 µm tall, with numerous granular contents and guttules. ***Basidiospores*** [60/3/2] 8–9.6(–10.0) × 6–7.8(–8.0) µm [*Q* = (1.12–)1.14–1.50(–1.60), *Q_m_* = 1.36 ± 0.10], ellipsoid to broadly ellipsoid, with a prominent apiculus, and several oleiferous guttules within the spores, pale yellow in KOH, inamyloid, thin-walled, smooth. ***Leptocystiada*** 45–69 × 2–4 µm, mostly clavate, hyaline, smooth, clamped, with branches. ***Contextual hyphae*** 4–6 µm in diameter, thin-walled, clamped.

**Figure 3.**
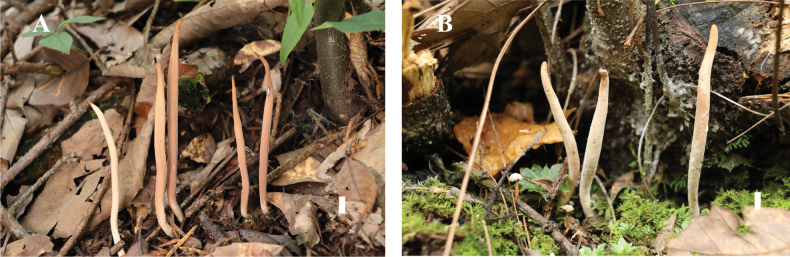
Basidiomata of *Clavariadelphus acuminatus*. A. MHHNU 12163; B. MHHNU 12164. Scale bars: 1 cm.

##### Ecological information and distribution.

Solitary, scattered or gregarious on the ground in mixed coniferous and broadleaved forest at elevations of 2300–2400 m. Southwestern China.

##### Additional material examined.

China • Yunnan Province, Gucheng District, Kainan Street, 26°77'41"N, 100°26'77"E, 2400 m asl., 9 September 2023 (MHHNU 12164).

##### Comments.

*Clavariadelphus acuminatus* is distinguishable from other Clavariadelphus species by the distinctive acuminate apex of the basidiomata. Morphologically, the taxon is similar to *C. khinganensis* in the color of the basidiomata, but the apex in *C. khinganensis* is obtuse or broadly rounded. In the phylogenetic analysis, *C. acuminatus* was the closest relative to C. *pseudoelongatus* with strong support (PP 1, BS 100%). Although the two species occupy a similar habitat, and have a subacute apex and flexuous basidiomata, in *C. acuminatus* the basidiomata have a brighter color and more strongly acute apex. *Clavariadelphus acuminatus* has pale to pale orange to apricot basidiomata, whereas *C. pseudoelongatus* has ecru-drab to light purple-drab basidiomata. In addition, microscopic features distinguish these species: *C. acuminatus* has smaller basidiospores (8–9.6 × 6–7.8 µm vs. 9.8–11.0 × 6.4–9.5 µm) and shorter basidia (75–99 × 8.5–13 µm vs. 81–113 × 8–13 µm). Thus, *C. acuminatus* and *C. pseudoelongatus* can be distinguished by the color of the basidiomata, and dimensions of the basidiospores and basidia.

**Figure 4.**
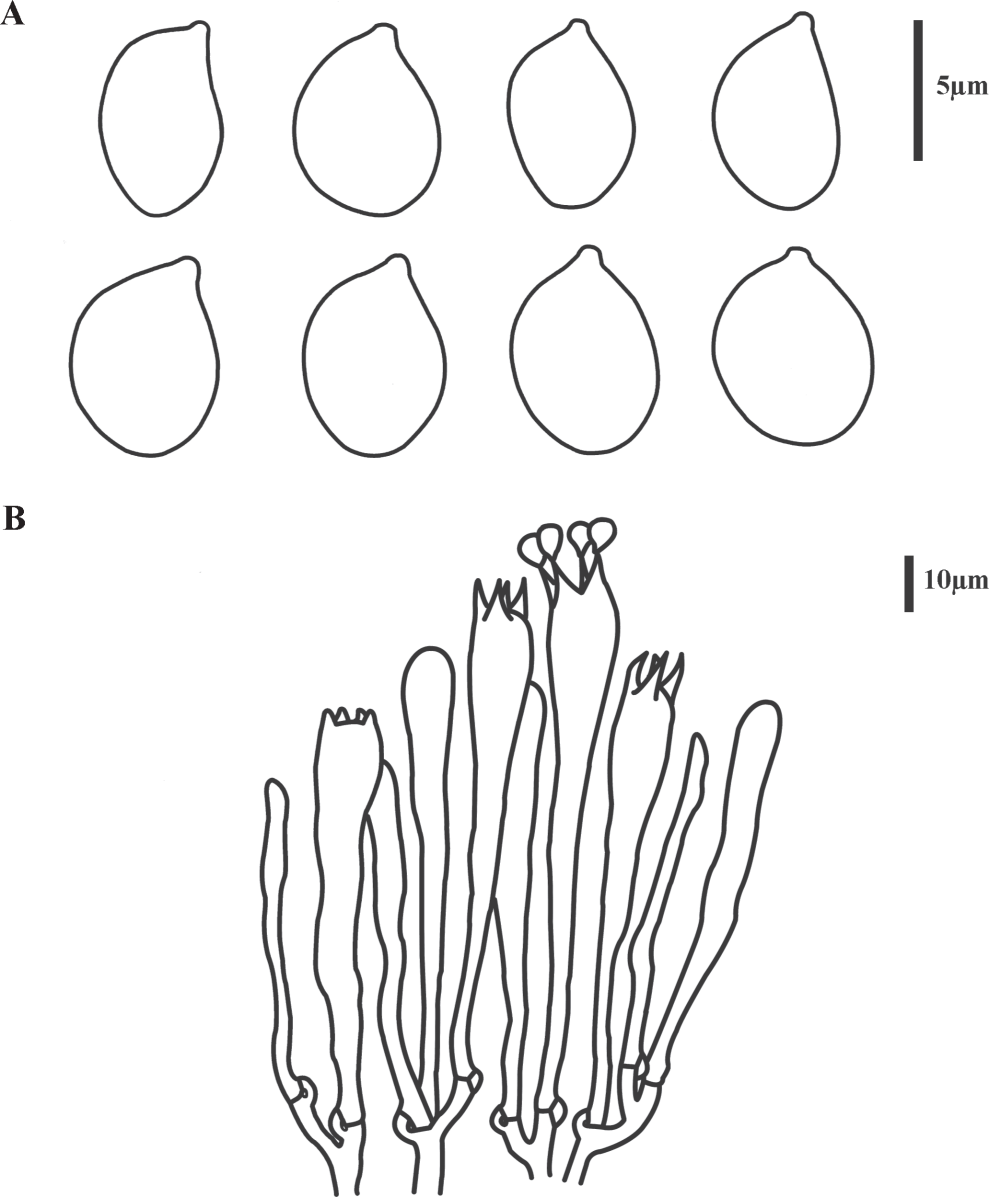
Microscopic features of *Clavariadelphus acuminatus* (MHHNU 12163). A. Basidiospores; B. Basidia.

#### ﻿ Clavariadelphus miniatus

Taxon classificationFungiGomphalesClavariadelphaceae

P. Zhang & M.J. Li sp. nov.

4DE11A67-94A9-50A3-AE57-20AD5B3C95F0

858804

##### Diagnosis.

Differs from other *Clavariadelphus* species in the small and pale yellow to salmon to purple-drab basidiomata with age.

##### Type.

China • Yunnan Province, Deqin Prefecture, 27°24'15"N, 98°57'55"E, 3750 m asl., 17 August 2018, leg. P. Zhang (holotype MHHNU 9952).

##### Etymology.

*Miniatus* (Latin) refers to the small basidiomata of this species.

##### Description.

***Basidiomata*** 4–10 cm tall, 0.2–0.5 cm in diameter, simple, narrowly cylindrical to clavate with age, slightly flexuous when young, occasionally branched; ***hymenium*** initially smooth, longitudinally rugose at maturity, pale yellow (2A3) to salmon (6A4) to purple-drab (11E5); apex fertile, smooth to rugose, obtuse to subacute, occasionally truncated with age, monochromatic with ***hymenium***; ***base*** terete, almost smooth, pale orange (5A3); ***context*** solid when young, gradually becoming soft and spongy with age. Odor and taste not recorded.

**Figure 5.**
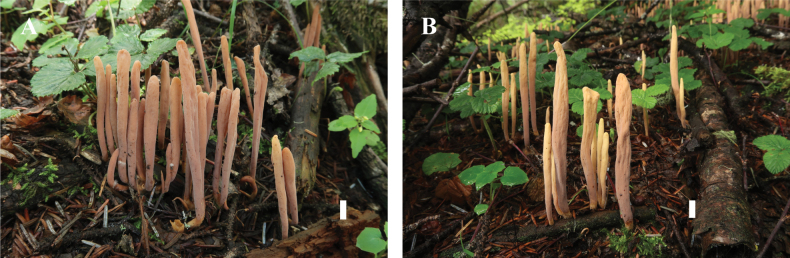
Basidiomata of *Clavariadelphus miniatus*. A. MHHNU 9952; B. MHHNU 12089. Scale bars: 1 cm.

***Hymenium*** extending over the apex of basidiomata, composed of basidia and leptocystidia. Basidia 72–120 × 10–13 µm, narrowly clavate, pale yellow in the KOH, smooth, thin-walled, clamped, 4 sterigmata (rarely 2), 6–12 µm tall, with numerous granular contents and guttules. ***Basidiospores*** [66/3/2] (16.5–) 18.0–26.6 (–27.0) × 4–6 (–7.2) µm [*Q* = (2.75–) 3.00–5.67 (6.50), *Q_m_* = 4.35 ± 0.79], ellipsoid to oblong, with a prominent apiculus, hyaline in the KOH, thin-walled, smooth, inamyloid. ***Leptocystida*** 30–82 × 3–4 µm, narrowly clavate, hyaline, smooth, clamped, with branches. ***Contextual hyphae*** 3–7 µm in diameter, thin-walled, clamped.

**Figure 6.**
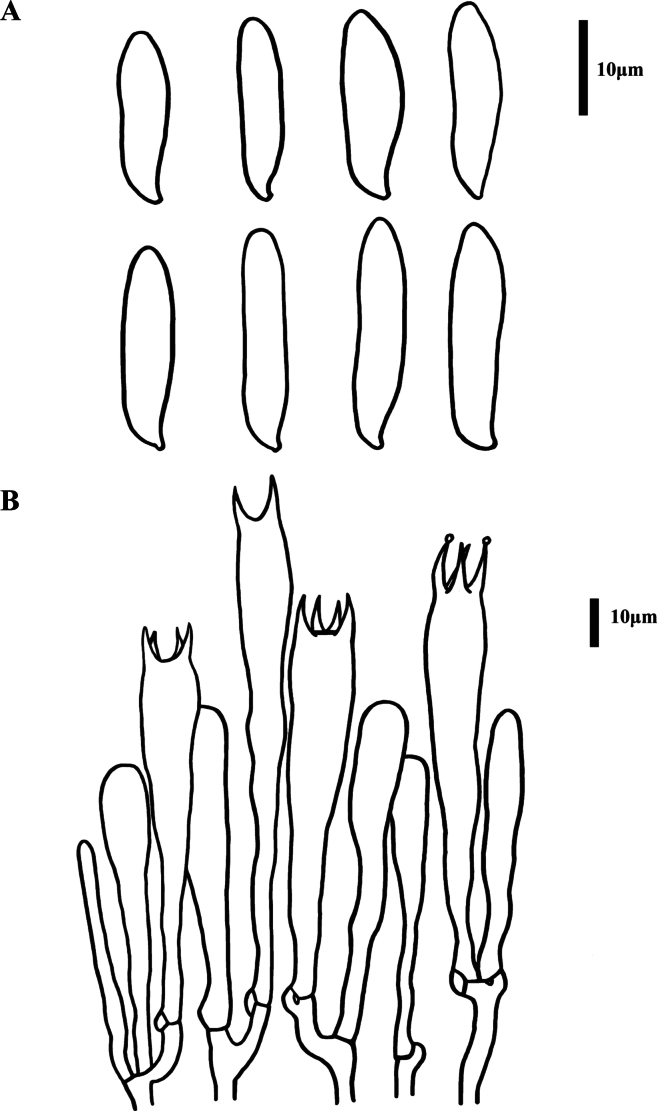
Microscopic features of *Clavariadelphus miniatus* (MHHNU 9952). A. Basidiospores; B. Basidia.

##### Ecological information and distribution.

Scattered or gregarious on the ground or on twigs in forest dominated by *Picea* in an elevational range of 3750–3800 m. Southwestern China.

##### Additional material examined.

China • Yunnan Province, Deqin Prefecture, Baima Snow Mountain, 28°15'28"N, 99°15'20"E, 3800 m asl., 19 August 2024 (MHHNU 12089).

##### Comments.

Unlike the other two new species identified in this study, *C. miniatus* is saprophytic and can be distinguished by the small size and pale yellow to salmon to purple-drab basidiomata. This taxon is confusable with *C. sachalinensis*. With regard to morphological characters and molecular sequence data, *C. miniatus* is similar to *C. sachalinensis*. Both species were resolved in a clade phylogenetically close to the two species of *Lentaria*, but their basidiospores are oblong, thin-walled, and hyaline in the KOH. *Clavariadelphus sachalinensis* has tawny or light walnut-brown to light brown basidiomata; thus, it is confusing to distinguish it from *C. miniatus* based on the color of the basidiomata alone. However, microscopic characteristics differ between *C. miniatus* and *C. sachalinensis*. Specifically, *C. miniatus* has longer basidiospores (18.0–26.6 × 4–6 µm vs. 21–24 × 4–6 µm) and basidia (72–120 × 10–13 µm vs. 65–105 × 8–12.5 µm).

#### ﻿ Clavariadelphus pseudoelongatus

Taxon classificationFungiGomphalesClavariadelphaceae

P. Zhang & Z. H. Chen sp. nov.

FAFBC858-991E-5809-BE82-442B80CBAA8E

858805

##### Diagnosis.

Differs from other *Clavariadelphus* species in having ecru-drab to light purple drab basidiomata with age, becoming cinnamon after cutting or bruising.

##### Type.

China • Yunnan Province, Shangri-La Prefecture, 27°13'13"N, 100°02'20"E, 3828 m asl., 26 August 2020, leg. P. Zhang (holotype MHHNU 32323).

##### Etymology.

*Pseudoelongatus* (Latin) refers to having longer basidiomata than other *Clavariadelphus* species, but differing from *C. elongatus*.

##### Description.

***Basidiomata*** 6–14 cm tall, 0.7–0.9 cm in diameter, simple, narrowly cylindrical to clavate in age, erect or slightly flexuous, occasionally branched; ***hymenium*** longitudinally apparent rugose, ecru-drab (5A2–4) to light purple drab (12D3–12D4), slowly stained cinnamon (6D6) after cutting or bruising; ***apex*** fertile, smooth to rugose, obtuse to subacute with age, monochromatic with ***hymenium***, rarely dark violet (16F8); ***base*** terete, almost smooth, white; ***context*** solid when young, gradually becoming soft and spongy in age. Odor and taste not recorded.

**Figure 7.**
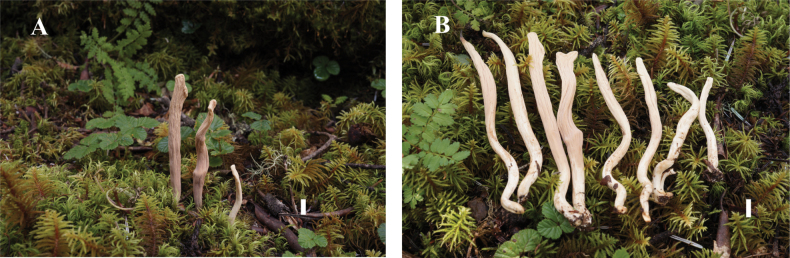
Basidiomata of *Clavariadelphus pseudoelongatus*. A, B. MHHNU 32323. Scale bars: 1 cm.

***Hymenium*** extending over the apex of the basidiomata, composed of basidia and leptocystidia. Basidia 81–113 × 8–13 µm, narrowly clavate, pale yellow in KOH, smooth, thin-walled, clamped, 4 sterigmata (rarely 2), 8–15 µm tall, with numerous granular contents and guttules. ***Basidiospores*** [60/3/2] (9.2–) 9.8–11.0 (–13.0) × (6.0–) 6.4–9.5 (–11.0) µm [*Q* = 1.16–1.55(–1.67), *Q_m_* = 1.31 ± 0.10], ellipsoid to broadly ellipsoid, with a prominent apiculus, and several oleiferous guttules within the spores, pale yellow in KOH, thin-walled, smooth, inamyloid. ***Leptocystida*** 31–71 × 2–6 µm, narrowly clavate, hyaline, smooth, clamped, with branches. ***Contextual hyphae*** 5–9 µm in diameter, thin-walled, clamped.

**Figure 8.**
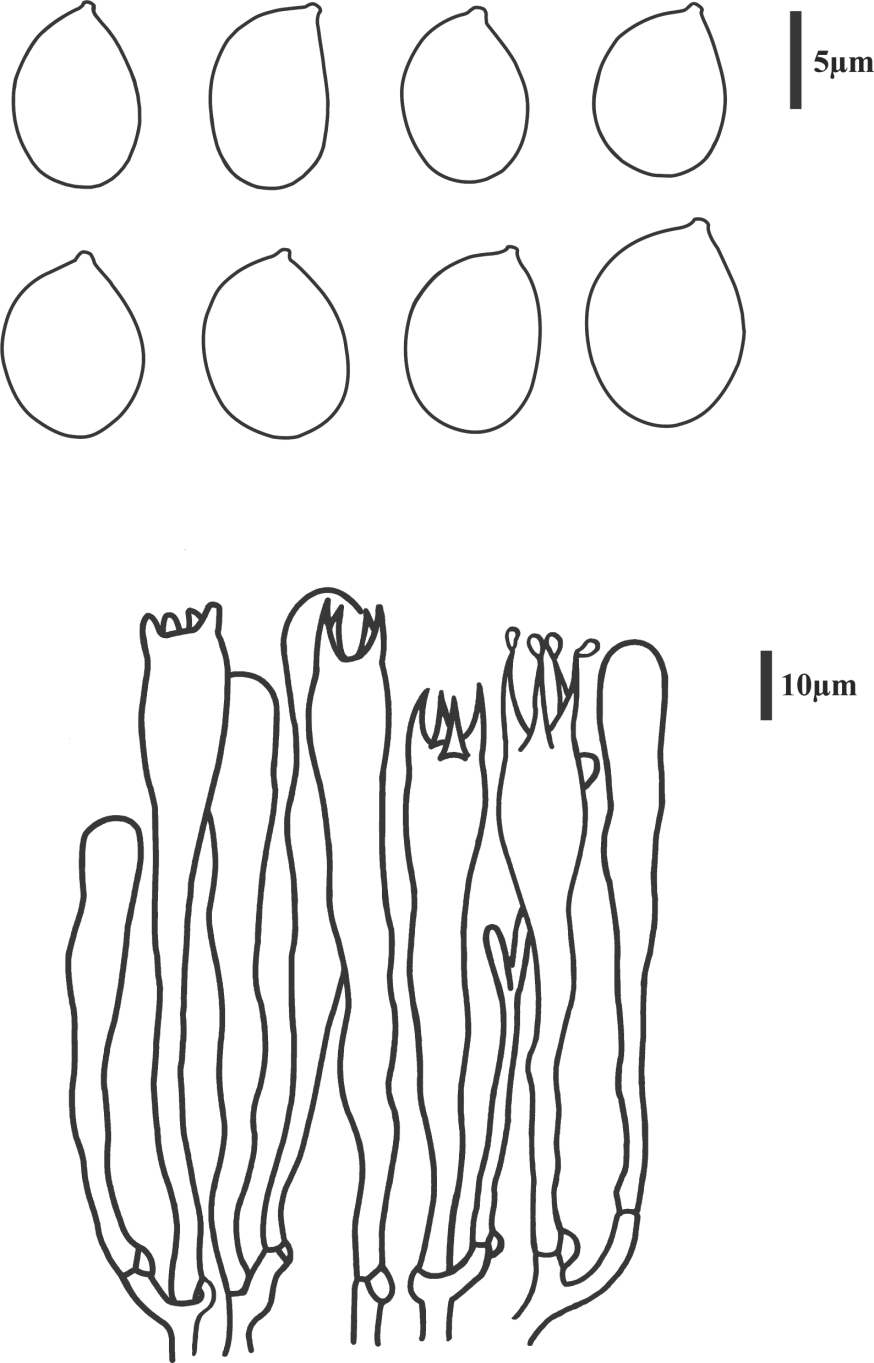
Microscopic features of *Clavariadelphus pseudoelongatus* (MHHNU 32323). A. Basidiospores; B. basidia.

##### Ecological information and distribution.

Scattered or gregarious on the ground in the coniferous and broadleaved mixed forest in an elevational range of 3800–3828 m. Southwestern China.

##### Additional material examined.

China • Yunnan Province, Shangri-La Prefecture, Qianhu mountain, 27°25'45"N, 99°40'55"E, 3800 m asl., 24 August 2024 (MHHNU 12123).

##### Comments.

*Clavariadelphus pseudoelongatus* can be distinguished by having longer and ecru-drab to light purple-drab basidiomata. It was initially misidentified as *C. elongatus* J. Khan, Sher & Khalid owing to the similar color of the basidiomata, which are light purple at maturity. In the phylogenetic analysis, *C. pseudoelongatus* and *C. elongatus* were respectively placed in different subclades based on molecular evidence. In addition, *C. pseudoelongatus* has broader basidiospores (9.8–11.0 × 6.4–9.5 µm vs. 9–11.0 × 5.7–7.4 µm) and larger basidia (81–113 × 8–13 µm vs. 75–95 × 6–10 µm) than *C. elongatus*.

## ﻿Discussion

Among *Clavariadelphus* species, certain species are saprophytic, while others are symbiotic (Hanif and Khalid 2013). In the present phylogenetic tree, Clade A+B and Clade C correspond respectively to symbiotic species and saprophytic species. Other than four *Clavariadelphus* species (*C. miniatus*, *C. sachalinensis*, *C. ligula*, and *C. griseoclavus*), 13 *Clavariadelphus* species that have an obtuse and fertile apex to the basidiomata were grouped in Clade A with strong support (PP 1, BS 98%). The remaining five *Clavariadelphus* species, which have a truncated and sterile apex, were grouped in Clade B with strong support (PP 1, BS 100%). *Clavariadelphus ligula* is unique in occupying saprophytic and symbiotic habitats. In the phylogenetic analysis, *C. ligula* was placed in the *Clavariadelphus* clade, but it formed an independent lineage separated from other *Clavariadelphus* species with strong support (PP 1, BS 100%). These results roughly indicate that this taxon may be transitional from saprophytic to symbiotic environments.

Although there are clear morphological differences between *Clavariadelphus* species and other members of Gomphales (Methven 1990), *Clavariadelphus* has a close relationship with other genera in Gomphales based on molecular phylogenetic analysis, such as *Ramaria* Holmskjöld, *Gomphus*, and *Lentaria* (Pine et al. 1999; Giachini et al. 2010). In particular, some species of *Clavariadelphus* have similar microscopic features and a saprophytic habitat comparable to *Lentaria* (Deng et al. 2020; Huang et al. 2020; Li et al. 2025), but few studies have closely investigated the relationship between *Clavariadelphus* and *Lentaria*. Therefore, a systematic analysis of the genetic relationship between these two genera is necessary. In the present research, all *Clavariadelphus* species were grouped in the same subclade except *C. ligula*. Meanwhile, two species of *Lentaria* (*L. byssiseda* and *L. patouillardii*) were embedded into *Clavariadelphus* species, but the statistical support was insufficient to corroborate a close relationship between *Clavariadelphus* and *Lentaria* owing to the limited data available. Thus, the relationship between the two genera requires further investigation.

The Hengduan Mountains region, a global hotspot of biodiversity, is an ideal habitat for fungi to inhabit, reproduce, and diversify (Mittermeier et al. 2005; Myers et al. 2000). Four newly identified species, *C. alpinus*, *C. amplus*, *C. aurantiacus*, and *C. tenuis*, were discovered in the Hengduan Mountains region in 2020, in addition to the three new species described in the present study. This region is rich in *Clavariadelphus* species diversity. In the future, we predict that a growing number of unnamed *Clavariadelphus* species will be found in the Hengduan Mountains region.

### ﻿Key to known *Clavariadelphus* species in China

**Table:** 

1	Basidiospores narrowly ellipsoid, *Q_m_* > 2	**2**
–	Basidiospores broadly ellipsoid to ellipsoid, *Q_m_* < 2	3
2	Basidiospores < 16.5 µm long, *Q_m_* < 4	***C. ligula***
–	Basidiospores > 16.5 µm long, *Q_m_* > 4	**4**
3	Basidiomata orange; apex sterile, truncate	**5**
–	Basidiomata without orange tinge; apex fertile, not truncate	**6**
4	Basidiomata pale yellow to salmon to purple-drab	***C. miniatus***
–	Basidiomata tawny or light walnut-brown to light brown	***C. sachalinensis***
5	Basidiomata apex 3–7.5 cm in diameter	**7**
–	Basidiomata apex < 2 cm in diameter	***C. gansuensis***
6	Basidiomata usually 20–30 cm high	**8**
–	Basidiomata usually < 20 cm high	**9**
7	Basidiomata yellow or light orange yellow	***C. aurantiacus***
–	Basidiomata pinkish orange or grayish orange	***C. amplus***
8	Basidiospores narrowly ellipsoid, basidiomata gray-purple	***C. elongatus***
–	Basidiospores broadly ellipsoid, basidiomata cinnamon	***C. yunnanensis***
9	Basidia clampless	***C. tenuis***
–	Basidia clamped	**10**
10	Basidiomata grayish red to pastel-red	***C. himalayensis***
–	Basidiomata gray or yellow, without red coloration	**11**
11	Basidiomata gray; basidiospores elliopsoid 10–11 × 5–6.5 µm, *Q*_m_ = 1.89	***C. griseoclavus***
–	Basidiomata yellow	**12**
12	Basidiomata yellow; basidiospores broadly ellipsoid, 7.8–9.6 × 5.5–7.4 µm, *Q*_m_ = 1.38	***C. alpinus***
–	Basidiomata pale yellow-brown; basidiospores narrowly ellipsoid 9.2–12.0 × 4.6–6 µm, *Q_m_* = 1.97	***C. khinganensis***
13	Basidiomata pale orange to apricot	***C. acuminatus***
–	Basidiomata ecru-drab to light purple-drab	***C. pseudoelongatus***

## Supplementary Material

XML Treatment for Clavariadelphus acuminatus

XML Treatment for Clavariadelphus miniatus

XML Treatment for Clavariadelphus pseudoelongatus

## ﻿Additional information

### Use of AI

No use of AI was reported.

### Author ORCIDs

Mei-jia Li https://orcid.org/0009-0000-0337-0283

Peng-tao Deng https://orcid.org/0000-0002-8755-7965

Zuo-hong Chen https://orcid.org/0000-0001-7359-0257

Ping Zhang https://orcid.org/0000-0002-8751-704X

### Data availability

All of the data that support the findings of this study are available in the main text.
